# A kidney transplant recipient with shingles and necrotizing bacterial superinfection: a case report

**DOI:** 10.3389/fmed.2026.1731010

**Published:** 2026-02-20

**Authors:** Anna Garstenauer, Raffael Scharinger, Christof Aigner, Florina Regele, Constantin Aschauer, Anselm Jorda, Georg Gelbenegger, Farsad Eskandary

**Affiliations:** 1 Division of Nephrology and Dialysis, Department of Medicine III, Medical University of Vienna, Vienna, Austria; 2 Department of Medicine I, Clinic Donaustadt, Vienna, Austria; 3 Department of Clinical Pharmacology, Medical University of Vienna, Vienna, Austria

**Keywords:** kidney tranplantation, necrotizing skin and soft tissue infection, *Pseudomonas aeruginosa*, superinfection, varicella zoster virus

## Abstract

We report a 45-year-old kidney transplant recipient who developed primary varicella zoster virus infection complicated by monomicrobial necrotizing skin and soft tissue infection caused by *Pseudomonas aeruginosa*. Progressive facial and oropharyngeal edema led to airway compromise requiring endotracheal intubation. Blood and wound cultures grew *Pseudomonas aeruginosa*, and targeted therapy with meropenem resulted in clinical improvement. The patient recovered with preserved allograft function but was left with residual facial nerve palsy. *Pseudomonas aeruginosa*–associated monomicrobial necrotizing skin and soft tissue infections are rare, occur predominantly in immunocompromised patients, and can be life-threatening.

## Case report/clinical picture

**FIGURE 1:**
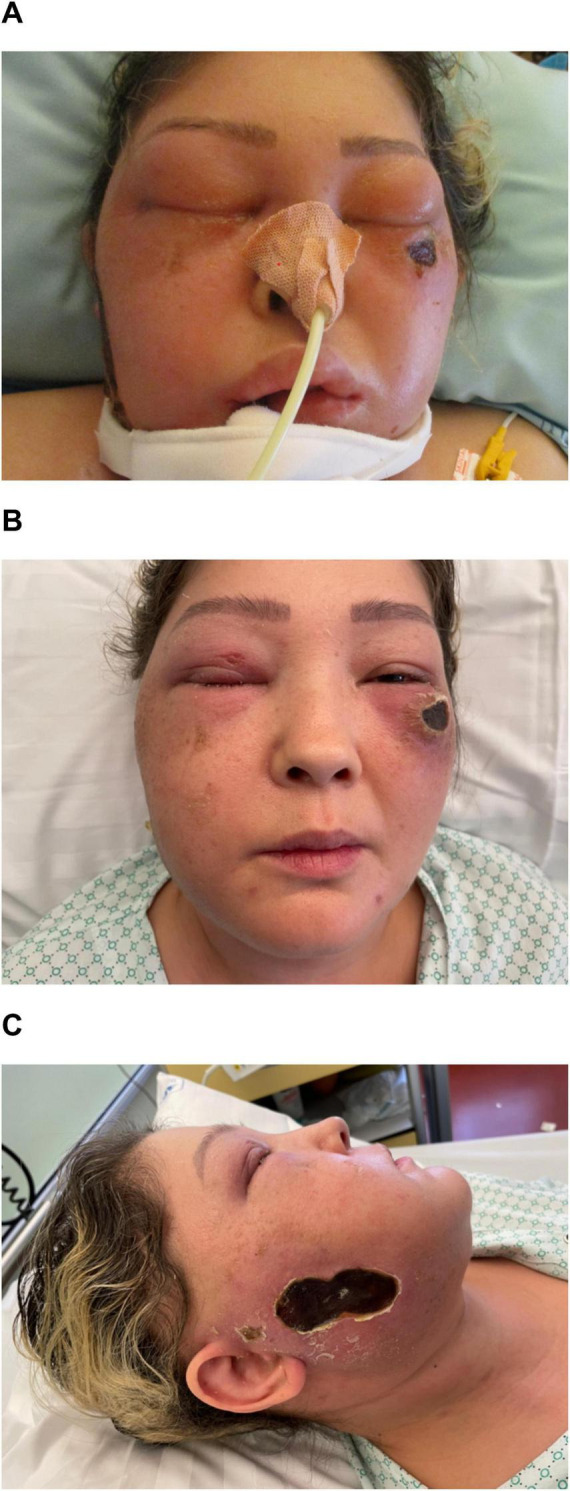
**(A)** Photo A was taken shortly after endotracheal intubation. A necrotising skin lesion can be seen under the left eye (and on the right cheek). **(B,C)** Photos B and C were taken soon after extubation on the medical ward, with some residual facial swelling still visible.

A 45-year-old woman who had undergone kidney transplantation 6 months earlier and was receiving triple immunosuppressive therapy with prednisolone, mycophenolate mofetil, and tacrolimus was transferred to our tertiary care center with progressive facial and oropharyngeal swelling accompanied by necrotizing facial ulcerations. One month earlier, she had been diagnosed with a primary varicella zoster virus (VZV) infection involving the chest, back, and left arm, which she had likely contracted from her daughter and was treated with oral valaciclovir. Her history of prior VZV infection or vaccination was unknown. Five days before admission, the lesions extended to the right side of her face, followed by progressive erythema and swelling. On admission, she was confused and anxious and was unable to open her eyes (Figure 1). Otorhinolaryngologic examination revealed marked edema of the epiglottis and edematous mucosa of the pharynx and nasopharynx, necessitating endotracheal intubation.

Laboratory investigations confirmed VZV infection [serum polymerase chain reaction (PCR) positive] and showed a markedly elevated C-reactive protein concentration of 28.4 mg/dL (reference range, <0.5 mg/dL). PCR testing of a swab from the necrotizing ulceration on the right side of the face was also positive for VZV, with a viral load of 2.9 × 104 copies/mL. To exclude central nervous system involvement, cranial computed tomography (CT) and lumbar puncture were performed. VZV PCR testing of the cerebrospinal fluid was negative. CT imaging showed no evidence of abscess formation or cerebral venous sinus thrombosis but demonstrated extensive soft tissue edema (Supplementary Figures 1, 2). At the time of presentation, surgical intervention was not indicated.

Mycophenolate mofetil was discontinued, and antiviral therapy was escalated to intravenous acyclovir (total treatment duration, 21 days). Empiric antimicrobial therapy with piperacillin–tazobactam initiated at the referring hospital was escalated to meropenem (total treatment duration, 21 days) and adjunctive topical fusidic acid was administered for 5 days. Both blood cultures and cultures from a swab of the facial lesions yielded *Pseudomonas aeruginosa*, which was susceptible to meropenem (Supplementary Tables 1, 2). Topical fusidic acid was replaced with bacitracin–neomycin and was administered for a total of 10 days. Microbiological testing for *Nocardia* species was negative.

The swelling decreased under targeted antimicrobial therapy, and the patient was extubated on day 10. She recovered well, with preserved kidney allograft function, but was left with a facial nerve palsy. Areas of necrotizing soft tissue persisted, and elective surgical management (scar revision and flap reconstruction) was planned.

*Pseudomonas aeruginosa*–associated monomicrobial necrotizing skin and soft tissue infections are rare but occur predominantly in patients with diabetes or immunocompromising conditions (1). In this patient, the presumed portal of entry was a disrupted facial skin barrier due to VZV lesions, allowing secondary invasion by *P. aeruginosa* and resulting in a potentially life-threatening superinfection.

## Data Availability

The datasets presented in this article are not readily available because this manuscript describes a case report. Requests to access the datasets should be directed to GG, georg.gelbenegger@meduniwien.ac.at.
