# Supplementary material for: A kidney transplant recipient with shingles and necrotizing bacterial superinfection: a case report
Source: Front Med (Lausanne). 2026 Feb 20;13:1731010. doi: 10.3389/fmed.2026.1731010 (PMC12963000; doi:10.3389/fmed.2026.1731010)
Supplement: Supplementary file 1 [file Data_Sheet_1.pdf]

Supplementary figure S1.

CT ON ADMISSION

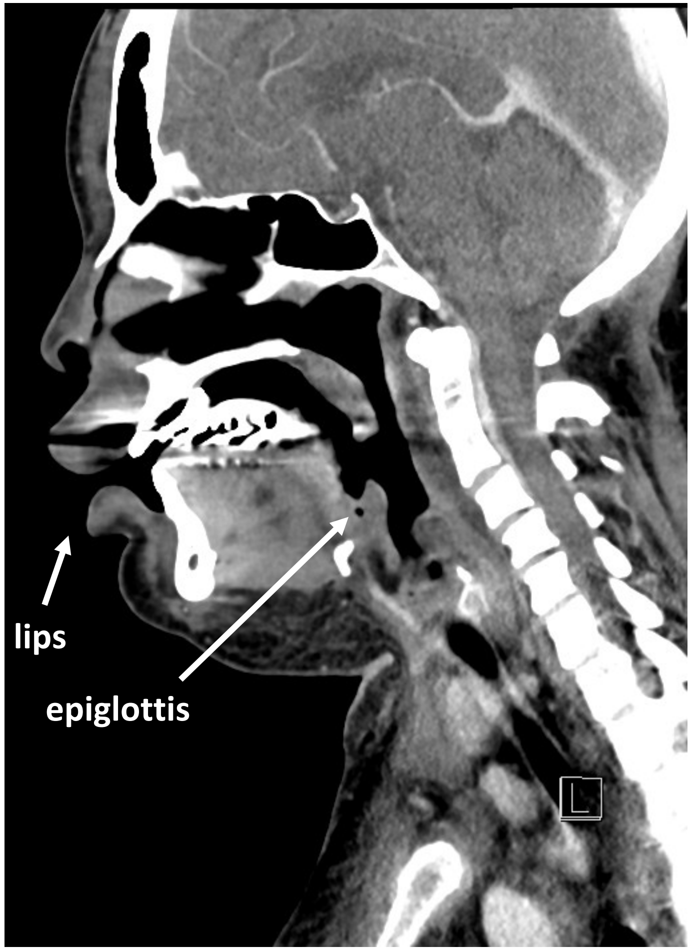

CT ONE WEEK AFTER EXTUBATION

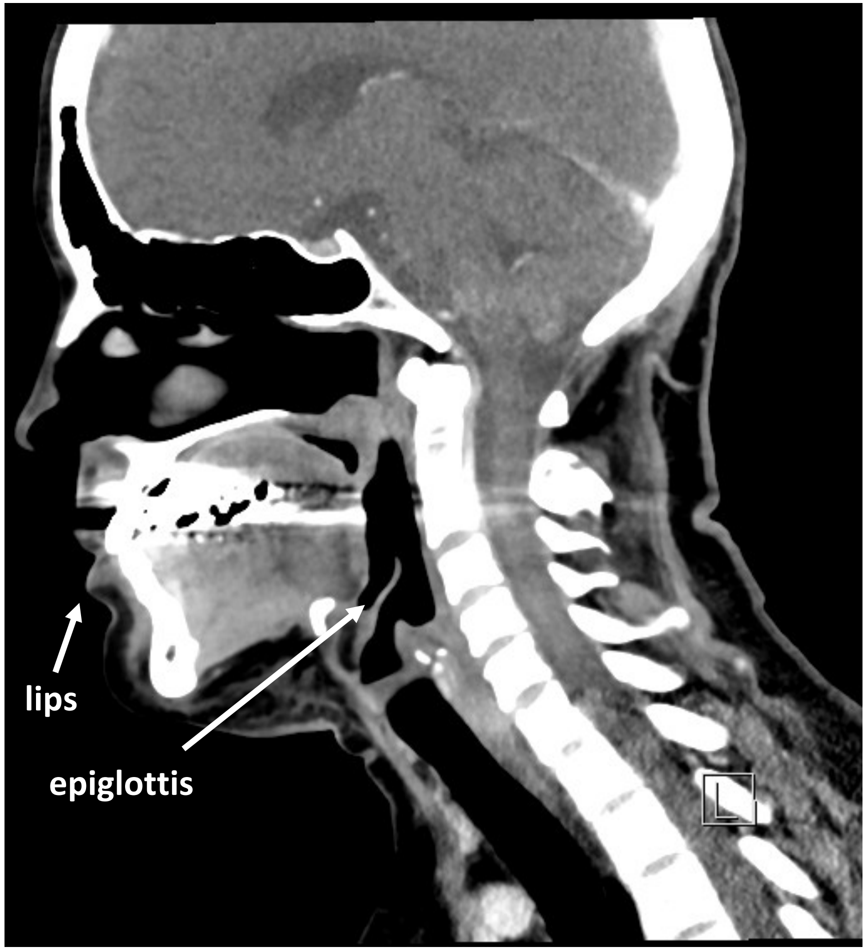

Supplementary figure S2.

CT ON ADMISSION

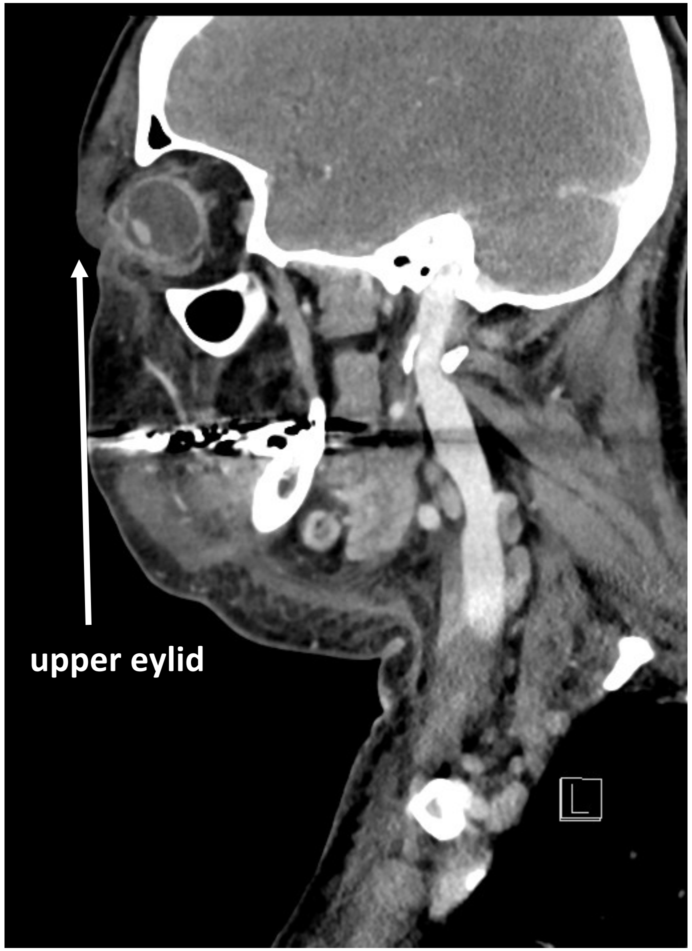

CT ONE WEEK AFTER EXTUBATION

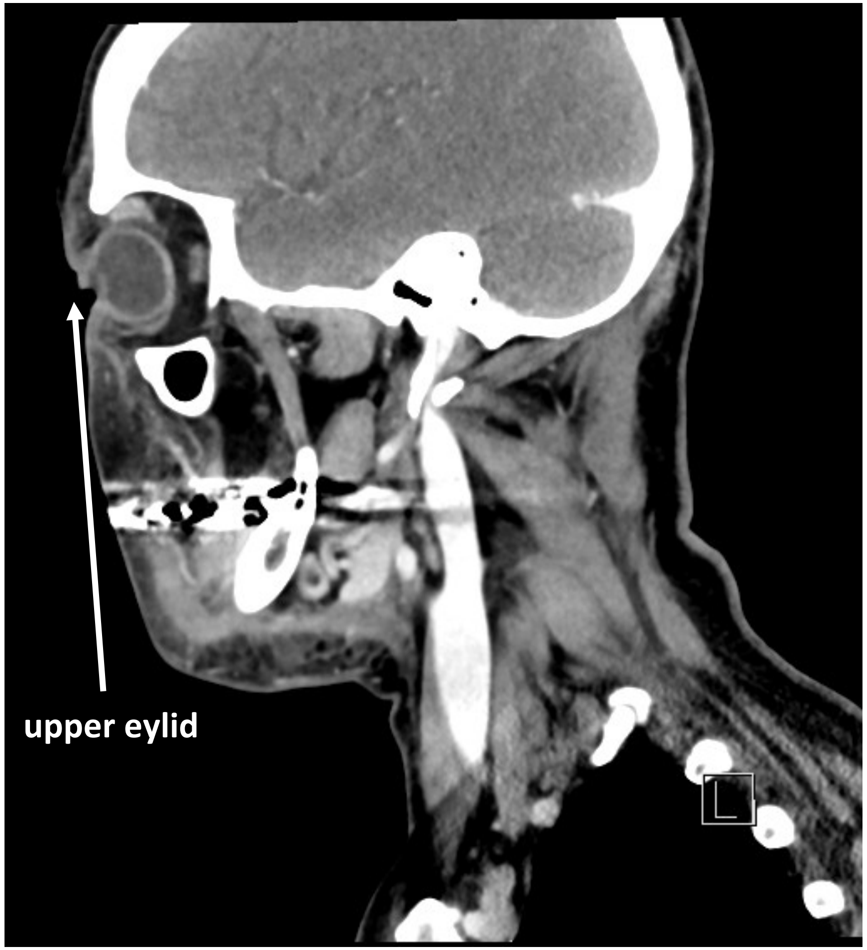

Supplementary tables S1 and S2. Antibiogram results

**Blood culture**	
Pseudomonas aeruginosa **(TTP 10h)**	
Piperacillin/Tazobactam	+/-
Ceftazidim/Avibactam	+
Ceftolozan/Tazobactam	+
Cefepim	+/-
Ceftazidim	+/-
Aztreonam	+/-
Meropenem	+
Imipenem	+/-
Ciprofloxacin	+/-
Amikacin	+/-
Tobramycin	+/-

TTP time to positivity

**Microbiological face swab**	
Pseudomonas aeruginosa	
Piperacillin/Tazobactam	+/-
Cefepim	+/-
Ceftazidim	+/-
Imipenem	+/-
Meropenem	+
Aztreonam	+/-
Amikacin	+
Tobramycin	+
